# 

**Published:** 1970-10

**Authors:** 


The
Bristol Medico-Chirurgical Journal
(Incorporating the Medical Journal of the South-West)
Established 1883

Vol. 85 (iv) OCTOBER 1970 No. 316

### BRISTOL MEDICO-CHIRURGICAL JOURNAL

Published Quarterly Annual Subscription 16/- Post Free

### BRISTOL MEDICO-CHIRURGICAL SOCIETY

**President 1969-70 : Dr. F. J. W. Lewis**
**President-elect : Dr. Beryl Corner.**
**Honorary Secretary : Dr. I. S. Bailey, 7 Percival Road, Bristol 8.**
**Honorary Treasurer : Dr. Frank Ross, Bristol Royal Infirmary, Bristol 2.**

The Society was founded in 1874. In 1883 publication of the Journal began and has continued quarterly since then. During the years 1949 to 1962 it appeared under the title of "The Medical Journal of the South West".

### THE BRISTOL MEDICO-CHIRURGICAL JOURNAL

### Editorial Committee

| | |
|---|---|
| Editor | Dr. N. J. Brown, Southmead Hospital, Bristol. |
| Assistant Editor | Mr. J. B. M. Roberts. |
| Members | Dr. M. B. Lennard.<br>Professor R. C. Wofinden.<br>Dr. D. W. Wright.<br>The Hon. Treasurer and Hon. Secretary of the Society. |
| Business Manager | Dr. P. J. F. Baskett, Frenchay Hospital, Bristol. |

### NOTICE TO CONTRIBUTORS

The Journal is especially concerned to provide a place for the recording of medical thought, interests, and practice in and around Bristol. It therefore welcomes articles that, as well as being of immediate interest to members, will document the local and contemporary medical scene for our successors.

Original articles are invited provided that they have not been published elsewhere. References in the text should be by author's name, with year of publication in parenthesis; they should be listed at the end in alphabetical order, the name of each journal or book being given in full — not abbreviated — and the title of the article added. Illustrations should be supplied ready for reproduction. Line drawings should be in Indian ink on firm white paper or board. The author will be informed if it is found necessary to ask him to pay for the making of blocks.

The following contributions will be especially welcome: notes of forthcoming events, meetings held, degrees conferred, staff appointments, honours and public appointments and other local news.

Twenty-five reprints of his article will be supplied free to each contributor. Quotations for larger quantities will be sent out with proofs and must be ordered when the corrected proofs are returned.

### Bristol Medico-Chirurgical Society

The eighth and final meeting of the session was held on 15th May, 1970, when Sir Vivian Fuchs, Director of the British Antarctic Survey, addressed the Society on "Human Endurance".

**PROVISIONAL PROGRAMME FOR THE SESSION 1970-71**

Wednesday, 14th October, 1970.
Dr. Beryl Corner. President's Address.

Wednesday, 11th November, 1970.
Mr. Justice Lawton. "Violence in Britain Today".

Wednesday, 9th December, 1970.
Clinical Meeting at Bristol Royal Hospital for Sick Children.

Wednesday, 13th January, 1971.
Dr. Glin Bennet, Mr. R. M. Pigott. "Plastic Surgery: Mind over Matter".

Wednesday, 10th February, 1971.
Mr. C. A. Brown. An ophthalmological topic.

Wednesday, 10th March, 1971.
Professor Otto Wolff. "Priorities in Paediatrics".

Wednesday, 14th April, 1971.
Symposium on the Problems of Drug Addiction arranged by Dr. A. M. G. Campbell.

Wednesday, 12th May, 1971.
Lord Cohen. "Medical Education and the G.M.C."

### News and Notes

Mr. A. E. S. Roberts, Librarian of the Bristol Medical School, retired in August, 1970. His paper on the growth and development of the Medical Library appears in this number of the Journal. The present high reputation of the Library has been achieved largely as a result of Mr. Roberts' energy and enthusiasm, and generations of students and teachers owe him an immense debt of gratitude for his kindly helpfulness and impressive efficiency. An authority on local medical history, his vast knowledge of the Medico-Chirurgical Society and of this Journal has been of inestimable value to secretaries and editors over many years. The Journal in particular is indebted to him for a very great deal of unobtrusive service, freely and voluntarily undertaken with genial good humour and without thought of reward. The Society expressed its thanks at its May meeting when a presentation was made to Mr. Roberts and the Journal would now like to place on record its own appreciation of his past efforts and to wish for him and Mrs. Roberts a long and happy retirement in the deightful part of Somerset in which they have chosen to live.

---

Dr. I. B. Sutherland has been appointed Senior Administrative Medical Officer to the South-western Regional Hospital Board. He succeeds Dr. J. S. Westwater to whom we extend our very best wishes on his retirement.

---

Mr. R. A. Sellwood, Ch.M., F.R.C.S., who graduated in Bristol in 1952, has been appointed to an Additional Chair of Surgery at Manchester University.

---

F.R.C.P. — I. R. S. Gordon; R. A. Craig
M.R.C.P. — R. V. Hague; C. J. Moran

---

**UNIVERSITY OF BRISTOL**

**Examination Results and Dissertations Approved**

Ph.D. — R. W. Else, R. C. Povey.

M.D. — R. J. Burwood, D. A. Griffiths, M. J. Lewis, J. B. Quartey-Papafio.

Ch.M. — K. E. F. Hobbs, P. J. B. Smith.

M.B., Ch.B. With honours — J. H. Gillmore, C. J. Tyrrell, Mary P. Waller, N. Puya. Pass — J. D. Anderson, C. M. Asplin, P. G. Baker, P. H. Baylis, S. J. Bentley, B. P. Bishop, A. C. Burden, J. H. Burton, Wendy A. Campion, Heather G. Clemett, S. Court, Susan K. Coward, T. J. David, G. J. Davies, M. G. Davies, R. Doherty, D. J. Dye, Margaret S. Early, P. D. Edwards, D. J. Elliott, B. J. Fairbrother, Gillian A. Gaydon, A. G. F. Gibson, N. J. Halliday, Sylvia M. Hawkes, Carol M. Herbert, A. K. Hodson, D. C. Hogg, D. R. H. Jones, F. D. J. Lane, Hilary Le Page, P. P. Lowe, Elizabeth M. Lumsden, V. C. Martin, P. J. Metcalf, Patricia L. Mitchell, S. Mobasseur, P. J. P. Morgan, T. H. Morris, C. A. Niles, O. B. Ogunnaike, R. Pearson, G. C. Pegg, Milda E. Simpson, P. A. Standing, P. G. N. Thornton, Ilona Tobey, J. P. Virjee, R. M. Wagner, Rosemary M. Westlake, C. M. Wulwik.

### INDEX TO VOLUME 85

### SUBJECTS

Africa, new medical school ... 89

Anticoagulants, drug interactions ... 101

Appointments ... 29, 62, 88, 110

Association of Clinical Pathologists ... 61

Breast carcinoma ... 75

Bristol Medico-Chirurgical Society ... 25, 60, 87, 110

Canynge Hall ... 11

Consultants, newly appointed ... 30, 62, 88

English, writing of ... 77

Exchange transfusion in anaemia ... 7

Insomnia, neuropsychiatric view of ... 45

Lesotho, medical officer in ... 69

Library, additions to ... 23, 40, 86

Library, medical school, history of ... 93

Liver transplantation ... 63

Lunatic asylum at Fishponds ... 41

Maternity services of Bristol ... 9

News and notes ... 29, 62, 88, 110

Obituaries — Bartlett, C. H. ... 56

— Brinckman, Winifred ... 56

— Neale, A. V. ... 106

— Ridgway, J. ... 82

— Wood, D. ... 83

Otology in South-west ... 49

Postgraduate education ... 26

Screening, population for disease ... 31, 37

Sociology, measurements in ... 81

Southmead Orchestral Society ... 19

South-west Ophthalmological Society ... 61

South-west Orthopaedic Club ... 27, 87

South-west Paediatric Club ... 60

Soviet Red Cross ... 53

This Week in Parliament ... 105

Treasurer, reflections of ... 17

Viper bites ... 3

Wisdom from the Past ... 21, 58, 84, 108

### AUTHORS

Benney, W. E. ... 69

Bradbeer, W. H. ... 49

Clutton-Brock, J. ... 81, 105

Cole, A. P. ... 7

Dent, N. A. ... 37

Hamblin, T. J. ... 101

Heney, N. M. ... 75

Johnson, M. G. ... 63

Macrae, J. ... 17

Moran, C. J. ... 3

Naish, J. M. ... 89

Phillips, H. T. ... 41

Raper, A. B. ... 77

Roberts, A. E. S. ... 93

Rowland, A. J. ... 31

Sluglett, J. ... 9

Warren, P. K. G. ... 45

Wofinden, R. C. ... 11

Wright, D. W. ... 53

